# Whither Enzymology in the Twenty First Century?

**DOI:** 10.3389/fchem.2016.00020

**Published:** 2016-04-22

**Authors:** E. N. G. Marsh

**Affiliations:** Departments of Chemistry and Biological Chemistry, University of MichiganAnn Arbor, MI, USA

**Keywords:** enzyme design, computational modeling, enzyme mechanisms, radical SAM enzymes, microbiome

## Introduction

This “grand challenge” article marks the creation of a new specialty section shared between *Frontiers in Chemistry* and *Frontiers in Molecular Biology* that is dedicated to enzymology and protein chemistry. My aim in this article is to provide a personal perspective on the challenges and opportunities for field of enzymology and the directions in which it could and should move in the coming years. I hope this will challenge the reader to think more deeply about this discipline and its broader impact on science. But before gazing into the future, it is worthwhile briefly reviewing how our field has evolved until now, as it is this perspective that frames our outlook.

*Whither Enzymology?* was the title of a symposium held in 1990 to honor the late Jeremy Knowles on the occasion of his Sixtieth birthday (Raines, 2008). Knowles was in the vanguard of a pioneering group of enzymologists who first employed the tools of physical organic chemistry to study enzyme reactions. In the 1970's his group was the first to determine the full set of elementary rate constants defining the free energy profile for an enzyme-catalyzed reaction—that of triose phosphate isomerase (Albery and Knowles, 1976). This information, put together with the crystal structure for this enzyme, allowed us to understand enzyme catalysis with the kind of precision previously only possible for simple organic molecules. Subsequently, his lab was among the first to use site-specific mutagenesis to make precisely defined changes to enzyme active sites, which combined with detailed measurements of their effect on catalysis, allowed the relationships between enzyme structure and mechanism to be elucidated.

Studies such as these blew away the “fluffy clouds” obscuring the view of earlier generations of enzymologists, revealing enzymes to be exquisitely evolved, highly complex and incredibly efficient organic catalysts, but nevertheless subject to, and understood through, the basic principles of chemistry—in Knowles' words: “not different, just better” (Knowles, 1991). This view has shaped enzymology for the past 25 years, and by extension our view of cellular biology. It directly underpins the burgeoning discipline of synthetic biology, a field that involves tinkering with enzymes on a scale unimaginable in Knowles' era.

The tools available to enzymologists have advanced immeasurably in the last 25 years, resulting in a paradigm shift in our approach to studying them. Not least, much of the drudgery (and perhaps also some of the fun!) of obtaining enzymes for study has been removed and the accessibility of enzymes has expanded enormously. The advent of genomic sequencing, combined with cheap, commercially available gene synthesis and protein affinity tags, allows one to select essentially any enzyme for study and produce large quantities of pure protein with minimal effort. What would once have taken many person years to accomplish can now be achieved in a few days. (Although some classes of proteins, e.g., integral membrane proteins still remain challenging to express.) Advances in structural biology mean that determining the 3-D structure of most enzymes, if not trivial, is now routine; indeed, thanks to structural proteomics initiatives, the structure is increasingly the first information to emerge for a new enzyme. Moreover, techniques for studying enzymes have become increasingly powerful: instrumentation more sensitive (for some techniques to the level of single molecules) and data analysis more sophisticated and rapid due to exponential increases in computing power.

With such powerful tools and so many enzymes to explore, it seems reasonable some 25+ years on from Knowles' apotheosis, once again to pose the question: whither enzymology? In attempting to answer this question, I propose the three “grand challenges” discussed below. The first two I have chosen because they fundamentally challenge our understanding of enzyme catalysis. The first challenge is to predict enzyme catalytic activity from structure and dynamic information; the second challenge to design enzymes from scratch that approach the activity of naturally evolved enzymes. The third challenges focuses on expanding the frontiers of enzymology by discovering new enzymes that catalyze new reactions.

## Challenge 1—predictive enzymology

Much of our effort continues to focus on understanding what makes enzymes “better” catalysts, with advances in techniques and methodology allowing us to tackle more complex enzymes and peer more deeply into the fundamental principles by which enzymes achieve such enormous rate enhancements (Wolfenden and Snider, 2001). But could enzymes perhaps also be different? For many enzymes we now have very detailed descriptions of their three-dimensional structures, the chemical identities of each intermediate in the reaction, the elementary rate constants connecting each step in the mechanism, the pK_*a*_s of active site residues etc. However, I would argue that we still don't fully understand how enzymes achieve such remarkable rate accelerations of the reactions they catalyze, often of 10^18^–fold, or even more.

Certainly we can appreciate the importance of contributions made by active site acids and bases (Toney and Kirsch, 1989), electrostatic interactions and transition state binding energy (Wolfenden, 1969) to catalysis. But when we attempt to quantify these various contributions to the reaction profile of a specific enzyme we nearly always come up short of catalytic power—usually by many orders of magnitude. For example, if we were to strictly apply the transition state binding formalism to explain rate enhancements, for many enzymes we would have to postulate transient, non-covalent interactions of remarkable strength—far tighter than even the strongest known stable interactions such as biotin-streptavidin.

Clearly the reductionist approach can only take us so far. What is missing from our understanding is how the large, complex and dynamic structure of the protein contributes to catalysis. Current models focus on the role of low frequency vibrational modes that are postulated promote catalysis by driving the substrates through the transition state to products (Kamerlin and Warshel, 2010; Kohen, 2015). Evidence for such “promoting vibrations” remains rather indirect as much of the vibrational action is thought to lie in the “terahertz gap,” a region of the electromagnetic spectrum that is extremely hard to access. Breakthroughs in experimental techniques may open up new opportunities to gain insight into catalysis. But it is worth emphasizing that to properly interpret such data for molecules as complex as enzymes it will still be necessary to understand the details of the chemical physics underpinning these vibrations and that for that foundational studies on small molecules will be essential.

Equally important are advances in computation that will allow us to accurately simulate enzyme reactions. We have seen great progress in the area of computational simulation over the last few decades, driven by improved methodology and, of course, the exponential increase in computing power. We are now approaching the point where, at least for simple enzymes, reaction trajectories and free energy profiles can be calculated that agree reasonably well with experimentally obtained data (Senn and Thiel, 2009; Van Der Kamp and Mulholland, 2013). One problem is that currently many simulations aim to reproduce prior experimental measurements, and it is unclear how much this prior knowledge influences the choice of parameters used to model the enzyme reaction.

Looking forward, an important goal will be to advance computational simulations to the point where they have predictive power rather simply being descriptive. Ideally, this would mean that starting from the 3-dimensional structure of an enzyme-substrate complex, and possibly including experimental information on protein dynamics, one could simulate the enzyme reaction at a level that accurately and reliably predicts parameters that can be experimentally determined, such as elementary rate constants for the reaction, and ultimately commonly measured kinetic parameters such as k_*cat*_ and K_*M*_. Among the challenges here are combining simulation techniques to deal with the many timescales that contribute to enzyme catalysis. Whereas chemical bonds are made and broken on the femtosecond timescale, i.e., within one bond vibration, protein conformational changes necessary for substrate binding and product release occur on the millisecond timescale. In between are events that are essential to catalysis such as coordinated protein motions and the formation of chemical intermediates that occur on a range of timescales from picoseconds to milliseconds. If we can fully recapitulate an enzyme reaction *in silico*, we might reasonably claim to have understood how an enzyme works.

## Challenge 2—designing enzymes from scratch

In contrast to the over 100 years spent studying natural enzymes, the design of new enzymes is very much a Twenty First century endeavor and one that has been greatly empowered by advances in computation. Enzyme design is important for two reasons. First, the ability to design highly efficient, extremely specific catalysts that function at room temperature and pressure under benign conditions has enormous practical applications; for example in replacing industrial processes that currently require high temperatures and pressures and/or expensive metal catalysts, or in the synthesis of fine chemicals containing complex functionality and many stereocenters. Second, enzyme design provides a rigorous test of our understanding of the principles of enzyme catalysis. If we *really* understand how enzymes work, we should be able to build them from scratch, incorporating the various “components” e.g., a substrate binding pocket, a catalytic general acid, stabilizing electrostatic interactions, etc., into an existing protein scaffold, or more ambitiously, into a protein specifically designed for the task.

Current *in silico* approaches to enzyme design attempt to achieve this by employing an “inside-out” approach, starting with a transition state for the desired reaction and then surrounding it with a constellation of active residues designed to bind the substrate and provide suitably placed catalytic residues (Mak and Siegel, 2014). Search algorithms are then used to identify a natural protein scaffold that can potentially accommodate the designed active site. The “theozyme” is then synthesized and usually subjected to optimization by several rounds of directed evolution to select enzymes with improved performance. Whether this last step really qualifies as “design” is left to the reader to decide!

So far, enzyme designers have, with good reason, tended to focus on rather simple reactions, for example the Kemp elimination and ester hydrolysis. The best designs exhibit some quite impressive k_*cat*_/k_*uncat*_ values of up to ~10^9^ after optimization (Blomberg et al., 2013), with the usual reference state for comparing the uncatalyzed reaction is water at pH 7. However, the average designed enzyme is still around 11 orders of magnitude worse than the “average” natural enzyme before optimization by evolution, improving to 9 orders of magnitude worse after optimization (Mak and Siegel, 2014).

An optimist might argue that, on a log scale, we are halfway to matching natural enzymes where k_*cat*_/k_*uncat*_ values of 10^18^ are typical. However, although water is an obvious choice for benchmarking enzyme reactions, it has been suggested that a more appropriate comparison would be a solvent such acetonitrile, to approximate the dielectric constant of a protein, containing acetate, an effective general base catalyst for the Kemp elimination (Korendovych and Degrado, 2014). This medium would represent a “non-designed” enzyme-like environment that design should improve upon. With this benchmark, the second order rate constant for the acetate-catalyzed Kemp elimination in acetonitrile can be compared with k_*cat*_/K_*m*_ for the designed enzyme. It would seem that we ought to be smarter than acetonitrile and acetate – but so far *no* designed Kemp eliminase has beaten this benchmark (!), although subsequent optimization by directed evolution has allowed designed enzymes to surpass this benchmark.

Humbling though the current performance of designed enzymes is, design is an activity where one learns as much, if not more, from carefully analyzing a failure as one does from success. Analysis of k_*cat*_ and K_*M*_ for designed enzymes suggests, unsurprisingly, that the challenge lies in catalysis, rather than binding, as K_*M*_ values are similar to natural enzymes whereas k_*cat*_ values are much, much smaller. However, this is a field that is still in relative infancy and there are good reasons to be optimistic that designed enzymes will match natural enzymes in the coming decades. For example, current computational design strategies use fairly basic models of transition state theory and don't include features such as protein dynamics and ground state destabilization that we know are important for catalysis. It is worth underscoring that the success of this grand challenge is intimately linked to success of the first challenge. This is especially true for the many multistep reactions that involve cofactors: if we don't fully understand the mechanism of an enzyme reaction, we are unlikely to succeed in designing such enzymes.

## Challenge 3—prospecting for new enzymes

In the process of understanding the fundamental biochemistry underpinning cellular metabolism and physiology we have discovered a remarkably diverse array of enzyme-catalyzed reactions. However, it is likely that we have only scratched the surface, and that there are many more fascinating enzymes waiting to be discovered. Much enzymological research has focused on human/mammalian metabolism, unsurprisingly given the importance of understanding enzymes, and the mechanisms by which they can be inhibited, for the design of effective drugs. But even in this intensely studied area, the functions of 20–25% of human protein-encoding genes remain completely unknown, and a good fraction of these are likely to encode new enzymes.

Compared with plants and microbes, animals are rather limited range of molecules they synthesize, relying on diet to obtain half the amino acids needed to build proteins and for the precursors to many essential enzyme cofactors (as vitamins). Therefore, the opportunities to discover new and useful enzymes are vastly greater among plants and microbes. These organisms synthesize an astonishing array of molecules, ranging from very simple organic molecules to natural products of remarkable complexity and a wide variety of polymeric materials. Microbes also colonize some of the least hospitable environments—from naturally occurring sulfurous hot springs to highly polluted sites where they can utilize some of the most persistent organic pollutants as carbon sources.

Where life exists under extreme conditions, there are certainly likely to be interesting and unusual enzymes waiting to be discovered, which, furthermore, may have important uses. For examples, consider viral reverse transcriptase (Gallo, 1971), bacterial restriction enzymes (Meselson and Yuan, 1968) and archaeal thermostable polymerases (Chien et al., 1976), which were all discovered long before their importance in revolutionizing molecular biology and medical research would be appreciated. CO_2_ fixation represents another highly timely example, as this step is generally considered to be the bottleneck in carbon assimilation by plants. Although the Calvin cycle is the best known and studied, six alternative microbial pathways for the fixation of CO_2_ have been identified so far, four of them in the last decade (Erb, 2011). The search for novel microbial pathways has resulted in the isolation of novel enzymes such as crotonyl-CoA carboxylase/reductase (Erb et al., 2009), the most efficient CO_2_-fixing enzyme described so far.

The importance of microbes, literally, much closer to our hearts has also recently been appreciated. The human microbiota represents a symbiotic collection of organisms that have recently been estimated to be present in similar numbers to our own cells (Sender et al., 2016), most of which reside in our gut. There is increasing evidence that these organisms play an important role in human health; they may, for example, process dietary molecules to increase their nutritional value, or render them less toxic, through metabolic pathways that we lack. For example there is recent evidence that changes in gut microbiota caused by the consumption of artificial sweeteners may induce glucose intolerance (Suez et al., 2014). The enzymology of the microbiome remains largely unexplored, but some of these undiscovered enzymes may prove vital for human health.

Finally, as an illustrative example of enzyme discovery, consider the radical SAM enzyme family. These enzymes catalyze a remarkably wide range of chemical transformations that are initiated by the one-electron reduction of S-adenosylmethionine by an iron-sulfur cluster. This generates a reactive adenosyl radical (and methionine) that subsequently abstracts a hydrogen atom from the substrate to initiate the catalytic cycle (Wang et al., 2014). Originally identified in studies on lysine fermentation in Clostridial bacteria (Chirpich et al., 1970), these enzymes, which are very oxygen sensitive, were for a long time thought to be isolated curiosities that were restricted to a few anaerobic fermentation pathways. However, it is now known that radical SAM enzymes play vital roles in the biosynthesis of many vitamins, in the modification of tRNA and rRNA, in post-translational modifications of proteins and in the biosynthesis of various secondary metabolites. Although almost all of the enzymes studied so far are microbial, it is very likely, despite their oxygen sensitivity, that radical SAM enzymes are also present in animals, including humans.

Our exploration of radical SAM enzymes was greatly aided by the identification of a characteristic sequence motif associated with the SAM-coordinating iron-sulfur cluster, which is unique to this family of enzymes. To date there are ~ 50,000 putative radical SAM sequences cataloged. A recent bioinformatics study was able to classify these into 50 different subgroups associated with a known or putative enzyme function. Yet this analysis also identified subgroups for which no known function exists and other putative radical SAM sequences that were unique. Furthermore, it is quite likely that even within the known functional subgroups that there are enzymes catalyzing unknown reactions. This suggests that we have much more to learn about the reactions catalyzed by radical SAM enzymes, and the same is undoubtedly true for many other enzyme families.

## Concluding remarks—the greater challenge

In conclusion, enzymology will not wither any time soon! We still have a lot to learn about enzymes as catalysts. But, going forward, what we do learn will be profoundly useful to society.

In closing, however, I want to mention one overarching challenge. For any discipline to thrive going forward it needs to recruit a diverse group of bright new minds. Cultivating the next generation of enzymologists requires engaging students at the undergraduate level, or even earlier. In contrast to the advances in the lab, enzymology in the classroom seems to have stood still. Comparing the chapters on proteins and enzymes in the biochemistry textbook I used as an undergraduate 30 years ago and the book I teach from today, the same three examples: hemoglobin, lysozyme and chymotrypsin, still dominate the pages. Of course, these were among the very first proteins to have their structures determined (Blake et al., 1965; Perutz, 1965; Matthews et al., 1967) and at one time these examples really did represent the cutting edge of our understanding of enzymes. However, other areas of biochemistry, for example transcription, translation and the role of RNA biology, appear to have done a much better job in remaining current and its unclear why enzymology seems to have been left behind. Updating the way we teach enzymology to reflect the discipline in the Twenty First century and, more broadly, recruiting people into science from a wider, more diverse cross-section of society is a challenge equal in importance to any of the scientific challenges discussed above.

## Author contributions

The author confirms being the sole contributor of this work and approved it for publication.

### Conflict of interest statement

The author declares that the research was conducted in the absence of any commercial or financial relationships that could be construed as a potential conflict of interest.
